# Editorial: Investigating social cognition and its contributions to promote bio-psycho-social function

**DOI:** 10.3389/fpsyg.2022.1064020

**Published:** 2022-11-30

**Authors:** Silvia Corbera, Satoru Ikezawa, Kee-Hong Choi

**Affiliations:** ^1^Department of Psychological Science, Central Connecticut State University, New Britain, CT, United States; ^2^Endowed Institute for Empowering Gifted Minds, Graduate School of Arts and Sciences, The University of Tokyo, Tokyo, Japan; ^3^School of Psychology, Korea University Mind Health Institute, Korea University, Seoul, South Korea

**Keywords:** social cognition, social functioning, quality of life, schizophrenia, neurocognition

Over the past years, there has been a great international emphasis in the fields of psychiatry, psychology, and social neuroscience to examine the social functional correlates of social cognition (Savla et al., 2013; Weinreb et al., 2022). It is well-known that social cognition (SC) plays an important independent role in influencing social functioning separate from neurocognition (Couture et al., 2006, 2011; Fett et al., 2011; Schmidt et al., 2011), but it is imperative to understand its concrete contributions with functioning, given its numerous distinct constructs and measures to assess them. The aim of the current topic in Frontiers was to aid in collecting new knowledge on this topic, by assembling both basic, translational, and applied on the topic of SC and its relationship with functioning. Social interactions occur embedded in a cultural and societal context which inherently influence the mental operations that are involved in processing social cognitive information. It is critical that to maximize knowledge in this area, to generate international cross-cultural research validating methods and treatment interventions. The present Research Topic revealed multiple international studies in the topic of SC, engaging multiple areas such as in the examining SC in non-clinical populations (Koo et al.; Lee and Choi), the neurobiological substrates of schizophrenia (Yagyu et al.), describing the outcomes of new pilot treatment interventions in schizophrenia (Haga et al.), developing new protocols in SC in schizophrenia (Palumbo et al.), providing basic evidence of gender differences in the relationship between neurocognition and social cognition in schizophrenia (Kubota et al.), and developing protocols for reviews and meta-analysis on psychotropic drugs and supplements on SC in schizophrenia (Yamada et al.).

To understand SC in non-clinical populations, Lee and Choi examined the potential influence of mothers' SC on their children's SC, social competence, and emotion regulation in a sample of immigrant mothers and their children residing in South Korea. They results showed that maternal SC skills, such as emotion recognition and theory of mind (ToM), contributed to children's emotion recognition and social competence. Since South Korea has rapidly transformed from an ethnically homogeneous country to a multicultural society due to an increase in migrant populations, the major strength of this study was focusing on immigrant mothers and their children. Further international collaborative studies that investigate maternal social-cognitive capacity with children's SC among multicultural families is warranted.

In the same line, in a 184 non-clinical sample, Koo et al. revealed that ToM mediated the relationship of hostile attribution bias with neurocognition, perspective taking and psychoticism. The authors suggested that in future studies, the mediating role of ToM should be examined in clinical populations, which would inform of treatment development, targeting ToM, and attributional bias.

Besides examining SC in non-clinical populations, it is important to understand the factors examining the deficits in SC in schizophrenia such as individual differences and sex differences. Kubota et al. provided evidence that revealed no sex differences in ToM or hostility bias in schizophrenia but showed that sex differences were observed in the relationship between neurocognition and ToM and neurocognition and hostility bias. This study was conducted with participants being within their first 5 years of their illness onset and therefore had the same length of disease duration. A separate study by Yagyu et al. examined the role of corollary discharge in people with or without schizophrenia by studying the differences in somatosensory evoked fields and somatosensory evoked potentials in the primary somatosensory area, elicited by stimuli produced either by the self or an external agent. Although the results should be replicated in a larger sample, contrary to the authors' hypothesis, there were no differences in corollary discharge in the primary somatosensory area between people with or without schizophrenia. However, positive symptoms of schizophrenia appeared to be associated with corollary discharge in the primary sensorimotor area. Additionally, this Frontiers topic included studies examining novel interventions targeted at improving social cognitive deficits. Haga et al. investigated Metacognitive Training (MCT) in an emergency psychiatric care setting. Twenty-four participants were randomly assigned to a group with MCT plus occupational therapy (OT + MCT) or to a group with OT only (OT-only). Results showed that the general psychopathology score was significantly reduced in the OT + MCT group than in the OT-only group and showed substantially lower readmission rates within 1 year in the OT + MCT than in the OT-only group. Although patients frequently voice the need for more psychosocial interventions in the emergency setting, and research is still scarce, this preliminary study with a small sample size, provided promising results for developing effective treatments. In the same line, but in a protocol stage, Palumbo et al., examined SC and narrative enhancement schizophrenia. The authors examined the feasibility and efficacy of the protocol and its effects to real-life functioning and generalization. They planned to deliver a baseline battery of assessments followed by either the SOcial Cognition Individualized Activities Lab program (SoCIAL) or Treatment as Usual, followed by a post-intervention assessment. This new promising intervention united both SC and narrative therapy with the aim to focus on improving social cognitive skills maximizing real-life long-term outcomes.

At the same time and revealing the common interest to examine interventions to improve SC, Yamada et al. developed a protocol for conducting a systematic review and meta-analysis on psychotropic drugs and supplements on social cognition in schizophrenia. This is the first protocol that by quantitatively measuring the efficacy of several pharmacological treatments, showed that it would be possible to compare the effects of psychotropic medications that have not yet been concurrently tested. These latter studies revealed important protocols that are expected to benefit the international knowledge of the efficacy of both behavioral and psychotropic treatments in social cognition.

To conclude, the studies included in this current topic of Frontiers of social cognition illustrated the diverse international research efforts conducted to study SC in multiple areas illustrating recently completed studies and newer developed protocols with promising outcomes.

## Author contributions

SC, SI, and K-HC contributed to being part of the editorial team for this topic and writing this editorial. SC edited it and submitted it. All authors contributed to the article and approved the submitted version.

## Conflict of interest

The authors declare that the research was conducted in the absence of any commercial or financial relationships that could be construed as a potential conflict of interest.

## Publisher's note

All claims expressed in this article are solely those of the authors and do not necessarily represent those of their affiliated organizations, or those of the publisher, the editors and the reviewers. Any product that may be evaluated in this article, or claim that may be made by its manufacturer, is not guaranteed or endorsed by the publisher.
